# Sexual and reproductive health and rights in humanitarian settings: a matter of life and death

**DOI:** 10.1186/s12978-023-01594-z

**Published:** 2023-03-10

**Authors:** Rose Mary Asong Tazinya, Ieman Mona El-Mowafi, Julia Marie Hajjar, Sanni Yaya

**Affiliations:** 1 Interdisciplinary School of Health Sciences, University of Ottawa, Ottawa, Canada; 2 NORImpact, Stavanger, Norway; 3 School of International Development and Global Studies, Faculty of Social Sciences, University of Ottawa, 120 University Private, Ottawa, ON K1N 6N5 Canada; 4 The George Institute for Global Health, Imperial College London, London, UK

**Keywords:** Sexual and reproductive health, Reproductive rights, Global Health, Humanitarian settings, Fragile settings

## Abstract

It is estimated that approximately 4.3 million sexually active persons worldwide will receive poor and/or limited access to Sexual and Reproductive Health (SRH) services in their lifetime. Globally, approximately 200 million women and girls still endure female genital cutting, 33,000 child marriages occur daily, and a myriad of Sexual and Reproductive Health and Rights (SRHR) agenda gaps continue to remain unaddressed. These gaps are particularly pertinent for women and girls in humanitarian settings where SRH conditions including gender-based violence, unsafe abortions, and poor obstetric care are among the leading causes of female morbidity and mortality. Notably, the past decade has featured a record high number of forcibly displaced persons globally since World War II and has led to over 160 million persons requiring humanitarian aid globally, 32 million of whom are women and girls of reproductive age. Inadequate SRH service delivery continues to persist in humanitarian settings, with basic services insufficient or inaccessible, putting women and girls at higher risk for increased morbidity and mortality. This record number of displaced persons and the continued gaps that remain unaddressed pertaining to SRH in humanitarian settings require renewed urgency to create upstream solutions to this complex issue. This commentary discusses the gaps in the holistic management of SRH in humanitarian settings, explores why these gaps persist, and addresses the unique cultural, environmental, and political conditions which contribute to continued SRH service delivery inadequacies and increased morbidity and mortality for women and girls.

## Introduction

It is estimated that about 4.3 million sexually active persons worldwide will receive poor and/or limited access to Sexual and Reproductive Health (SRH) services in their lifetime [1]. Up until the end of June 2020, 200 million women and girls globally still endured female genital cutting along with the severe lifelong physical and psychological consequences that accompany this practice [2]. In addition, approximately 33,000 child marriages still occur daily, 25 million unsafe abortions occur annually, and a host of other unresolved Sexual and Reproductive Health and Rights (SRHR) agenda gaps remain unaddressed [1, 2]. These gaps are even greater among women and girls in humanitarian settings where SRH conditions like gender-based violence, unsafe abortions, and poor obstetric care are among the leading causes of female morbidity and mortality [3–5]. Indeed, women and girls in humanitarian settings continue to be deprived of basic SRHR resulting in devastating consequences [2, 6]. Humanitarian emergencies are known to be “a complex mix of occurrences that may result from natural forces (extreme weather or geological activity) or human activities (conflict, social upheaval, and environmental degradation)” [7].

The past decade has recorded the highest numbers of forcibly displaced persons globally since World War II; from 51.2 million in 2013 [8], to 65.6 million by the end of 2016, and to 79.5 million in 2019 [9]. These numbers, which continue to rise, have led to over 160 million persons requiring humanitarian aid globally, which includes over 32 million women and girls of reproductive age [10]. Moreover, the number of these persons returning home is greatly declining [1, 9]. This rapid increase is partly due to the high number of natural disasters, with approximately 340 occurring annually that affect over 200 million people [8, 11]. Additionally, displacements also result from armed and political conflicts and from conflicts over scarce resources, environmental stress, and human rights violations [1, 12]. These factors have consequently increased the burden on humanitarian settings both internally (forceful displacements from homes but persons remain within their country) and externally (where the persons displaced move out of their country) [11].

A 2018 UNFPA report confirmed that over 500 women and girls die daily from pregnancy and childbirth-related complications in humanitarian settings [6]. In Iraq, about 28% of girls were married before the age of 18 during the eighth year of the humanitarian crisis [13] and at least 21 young girls between 10 and 14 years were raped daily during the internal conflict in Columbia in 2018 [1]. Overall, more than 50% of maternal and 45% of neonatal deaths in the world occur in humanitarian settings [14, 15], indicating the urgent need to address and improve access to quality SRH services in humanitarian settings to protect women, children, and communities.

SRH is defined as “a state of physical, emotional, mental, and social well-being in relation to all aspects of sexuality and reproduction, not merely the absence of disease, dysfunction, or infirmity” [13]. To enable sustainable SRH, the sexual rights of all must be achieved. This includes the rights of women and girls and other vulnerable populations, especially those in humanitarian settings [16]. The International Conference on Population and Development (ICPD) defined the human rights of women as “their right to have control over and decide freely and responsibly on matters related to their sexuality free of coercion, discrimination, and violence.” [1, 16]. These definitions integrate SRH and basic human rights making it a fundamental obligation for everyone to be entitled to the best available standard of care for SRHR [17].

Despite the interventions in place, evidence suggests that disparities continue to persist between the SRHR agenda and current outcomes, particularly in humanitarian settings [13]. Understanding the root causes of these gaps is essential as this will enable the development of improved approaches to achieve upstream population-based decisions that will provide impactful and sustainable solutions [18]. This commentary will discuss the gaps in the holistic management of SRHR in humanitarian settings, will explore why these gaps persist and will discuss how they expose women and girls to increased morbidity and mortality.

### Impact of humanitarian contexts on SRHR risks, morbidity and mortality, access to care, and outcomes

Although women and girls in displaced and refugee settings often experience a greater need for SRH services they are often neglected, resulting in limited access to basic SRH services, thereby increasing their risk of morbidity and mortality [4, 5]. Inadequate SRH service delivery in humanitarian settings is due to three main factors. First, the systems and institutions that normally provide SRHR services are weakened or destroyed by the resulting crisis [19, 20] and second, victims have limited access to financial and material resources and suffer from stigma and discrimination [19]. Third, persons in these settings often lack information and knowledge about their rights and services linked to SRH which usually affects their power to negotiate and make decisions about their own health [13, 21].

At the community level, displacement causes family and community bonds to dissociate as family members get separated, and local and social norms are no longer respected [20]. Adolescent girls and young women are particularly impacted [22, 23] primarily because they constitute the highest proportion of those affected by conflicts and migration globally, which exposes them to the inherent vulnerabilities in humanitarian settings including sexual assault, stigma and discrimination, low access to quality health services and inadequate resources [24, 25]. Also, in most settings (including vulnerable settings), adolescent girls and young women are more deprived of family roles and resources, such as education and finance, and have higher exposure to other forms of violation including child, early and forced marriage, female genital mutilation, and honor killing compared to their male peers [20, 23]. This results in an increased risk of sexual and gender-based violence (GBV), sexually transmitted infections (STIs) including Human Immunodeficiency Virus (HIV), subsequent unwanted pregnancies and unsafe abortions, and birth-related complications, which inevitably lead to maternal deaths that could have otherwise been preventable [22, 25, 25, 26]. Studies have confirmed at least one in five women are victims of sexual violence in humanitarian settings [14, 27]. Victims of GBV often suffer and die due to related complications including injury and ill health from STIs like HIV, murder from practices like honor killings, and suicide from social and psychological trauma [7, 28]. There is evidence of high rates of mortality resulting from GBV. For example, in the Burmese refugee population in Thailand, over 5 million women die from honor killings annually and 2 out of 3 victims of suicide are women, with most resulting from domestic violence and rape [7]. Additionally, young girls who are involved in child marriage have an increased risk of experiencing domestic violence and early pregnancy, and most end up having unsafe abortions with associated complications which increases their risk of mortality [13].

In the context of healthcare service delivery, basic services such as safe abortion, family planning, and prenatal and postnatal care are usually not sufficiently provided or are inaccessible [1, 26, 29]. Health supplies including medication and equipment are largely insufficient with very few trained health personnel [4, 29] aware of the specific needs of this vulnerable group [13]. Moreover, healthcare providers in these settings are often working under dire conditions with very limited essential material, have low salary rates that are paid irregularly, [30, 31] and are sometimes victims of violence and harassment which leads to psychosocial discomfort and eventual low performance at work [31]. This leads to poor SRHR services which ultimately exposes women to further negative SRH outcomes. A study in Pakistan in 2011 revealed that women who gave birth in relief camps during the flood had no skilled birth attendants present, used unhygienic birth stations, and had poor postnatal services which increased the risk of mortality of these women and their newborns [4]. Another study in Africa confirmed only 5 out of 63 facilities in a humanitarian setting provided adequate obstetric and newborn care [14]. Even though unsafe abortions can constitute up to 50% of maternal deaths in humanitarian settings [13, 14], studies have confirmed the continued absence of safe abortion services in most health facilities in these localities [14], illustrating a significant gap that must be addressed urgently.

### Persistent SRHR gaps in humanitarian settings

Despite the progress in SRHR globally and the evidence that access to these services improves well-being, saves lives, and constitutes a basic human right, SRH services for persons in humanitarian settings continue to remain highly suboptimal [14, 22, 32]. The root causes for poor SRHR are associated with the unique cultural, environmental, and political conditions in these settings [18].

#### Socio-cultural factors

Displaced persons are often discriminated against due to their vulnerable status, their sexual orientation, and unintended premarital pregnancies which negatively impact their SRHR [18]. Several studies have reported the judgmental attitude and rudeness of healthcare providers providing SRH services in these settings which further hinders access to available SRH services by refugees and migrants as most of them shy away [33–35]. Cultural and language differences between migrants and healthcare workers also interfere with proper SRH services, as there is miscommunication and misunderstanding which sometimes causes misdiagnosis and eventual poor treatment [18]. Some nations and societies do not tolerate SRH practices such as abortion and the use of contraception. These topics thus remain taboo, and these attitudes make accessing SRHR services difficult even in cases where these interventions would benefit the health of the individual [33, 36]. There continues to be misunderstanding and misconception surrounding services such as contraception, with many individuals believing it causes infertility and cancer [37]. Other cultural and religious beliefs that prohibit premarital sex [21] and forbid others, especially men, from touching married women (even when they need health services) further hinder women’s access to SRHR services, promotes stigma and discrimination, and exposes women and girls to increased risk of mortality given that SRH services are not being used and complications are thus left unattended [37].

#### Environmental factors

Emerging threats and natural disasters continue to negatively impact SRHR services in humanitarian settings due to the increase in the number of displaced persons, which subsequently increases the burden placed on humanitarian settings. Also, resources for SRH are further limited as they are shifted away from vital SRH services to respond to the current crisis at hand [1, 14, 38]. For example, in Sierra Leone during the Ebola outbreak, there was a drastic drop in ante-natal care and family planning services in the camps as resources were shifted to handle the current crisis. This consequently led to over 3600 additional maternal, stillborn, and neonatal deaths between 2014 and 2015 [39]. Moreover, SRH access is even further reduced during disasters and crises due to lockdowns and other crisis-related restrictions like reduced mobility and social distancing that limits access to health facilities [40, 41]. For instance, during the Ebola lockdown, maternal mortality increased by 75% due to the inability to access SRHR information, services like safe abortion, and modern contraception, which consequently increased the rates of unintended pregnancies and left many lives at risk [41]. Similar findings occurred during the COVID-19 pandemic, where SRHR needs were highly neglected leaving more people exposed to gender-based violence that consequently increased morbidity and mortality [42].

Humanitarian settings are noted for poor living and working conditions and poor access to SRH which is sometimes life-threatening [22, 38, 43]. These locations, which are usually remote with limited access to proper healthcare facilities [44], have most of their inhabitants living in poor and illegal conditions making it more difficult for them to move to access better services elsewhere [22]. Moreover, most of the health services are delivered with payments out of pocket, which most of these inhabitants cannot afford. Consequently, the cost of important SRH services renders this important care inaccessible for these vulnerable populations, thereby increasing the risk of unintended pregnancies, unsafe abortions, and maternal deaths, which are ultimately preventable in these situations [22].

#### Political factors

Although efforts have been made by international, regional, and national agencies to improve SRHR service delivery in humanitarian settings [45], the geopolitical and historical contexts have not enabled horizontal collaborations. Significant upstream structures including neoliberalism and the humanitarian structure continue to cause friction at the national level leading to inadequate resource availability and poor SRH services [1]. Other factors include the limited capacity of global humanitarian agencies which are strained due to the rise in the number of displaced persons [1], and the neglect of some groups and services by rescue initiatives and international agencies [13]. For instance, adolescents are neither catered for by GBV services nor child protection services, which causes unaddressed violations like child marriage in this group [13, 25]. The non-inclusion of abortion services in the Minimum Initial Service Package (MISP) for SRH even for rape victims fails to address these particular needs for women and girls in crisis settings as abortion services are not offered when needed [13]. Moreover, most host countries of displaced persons do not offer sustainable solutions to ensure that proper SRH services and protection are offered for refugees [22]. Furthermore, available programs are lacking sufficient funding, proper program evaluation, and adequate implementation services to measure and monitor the effectiveness of services in these populations [14, 46].

## Conclusion

Conditions related to SRHR continue to be the leading cause of death and suffering among women of childbearing age in humanitarian settings globally [3]. The increasing number of persons requiring humanitarian support, poor health infrastructure, and insufficient policies to guide and supervise SRHR interventions and regulations in these settings continue to be major setbacks to the provision of holistic care. These gaps continue to expose vulnerable women and girls to a higher risk of mortality and related complications which requires urgent action to ensure that holistic care is offered in an environmentally relevant and upstream manner. To achieve sustainable and universal access to SRH, more investment in comprehensive and integrated services to target hard-to-reach areas and poorer settings must be employed [47, 48]. This should not be limited to the provision of comprehensive services but should also involve the provision of legal and policy mechanisms and proper evaluation to guide the implementation of these services [34, 46]. A clear and in-depth understanding of regulations guiding the SRHR of these populations by both lived-experience insights gained directly from inhabitants in addition to the insight provided by service providers is also paramount to integrating both perspectives to better understand how to address these continuing gaps that prevent women and girls from achieving optimal SRH [5, 14]. Therefore, highly active multi-sectoral collaboration is required to provide high-quality and accountable services [49] and to recognize that SRHR constitutes a basic human right that does not need to be violated [22, 50].

## Data Availability

Not applicable.
